# Nanomedicine Innovations for Lung Cancer Diagnosis and Therapy

**DOI:** 10.1021/acsami.4c16840

**Published:** 2024-12-31

**Authors:** Valéria Maria de Oliveira Cardoso, Maria Julia Bistaffa, Raquel González Sterman, Lorena Leticia Peixoto de Lima, Gustavo Silveira Toldo, Juliana Cancino-Bernardi, Valtencir Zucolotto

**Affiliations:** † Nanomedicine and Nanotoxicology Group, São Carlos Institute of Physics, University of São Paulo, 13560-970 São Carlos, São Paulo, Brazil; ‡ Chemistry Department, Laboratory in Bioanalytical of Nanosystems, Faculty of Philosophy, Sciences and Letters of Ribeirão Preto, University of São Paulo, 14040-901 Ribeirão Preto, São Paulo, Brazil; § Comprehensive Center for Precision Oncology, C2PO, University of São Paulo, São Paulo 01246-000, Brazil

**Keywords:** lung cancer, nanotechnology, nanomedicine, physiological barriers, nanocarriers, diagnoses and treatment of lung cancer

## Abstract

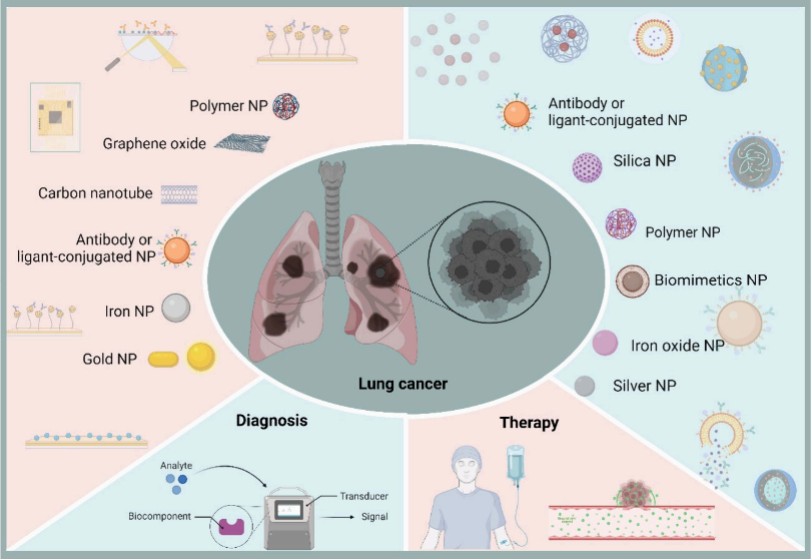

Lung cancer remains a challenge within the realm of oncology. Characterized by late-stage diagnosis and resistance to conventional treatments, the currently available therapeutic strategies encompass surgery, radiotherapy, chemotherapy, immunotherapy, and biological therapy; however, overall patient survival remains suboptimal. Nanotechnology has ushered in a new era by offering innovative nanomaterials with the potential to precisely target cancer cells while sparing healthy tissues. It holds the potential to reshape the landscape of cancer management, offering hope for patients and clinicians. The assessment of these nanotechnologies follows a rigorous evaluation process similar to that applied to chemical drugs, which includes considerations of their pharmacokinetics, pharmacodynamics, toxicology, and clinical effectiveness. However, because of the characteristics of nanoparticles, standard toxicological tests require modifications to accommodate their unique characteristics. Effective therapeutic strategies demand a profound understanding of the disease and consideration of clinical outcomes, physicochemical attributes of nanomaterials, nanobiointeractions, nanotoxicity, and regulatory compliance to ensure patient safety. This review explores the promise of nanomedicine in lung cancer treatment by capitalizing on its unique physicochemical properties. We address the multifaceted challenges of lung cancer and its tumor microenvironment and provide an overview of recent developments in nanoplatforms for early diagnosis and treatment that can enhance patient outcomes and overall quality of life.

## 1 Introduction

**Figure 1:**
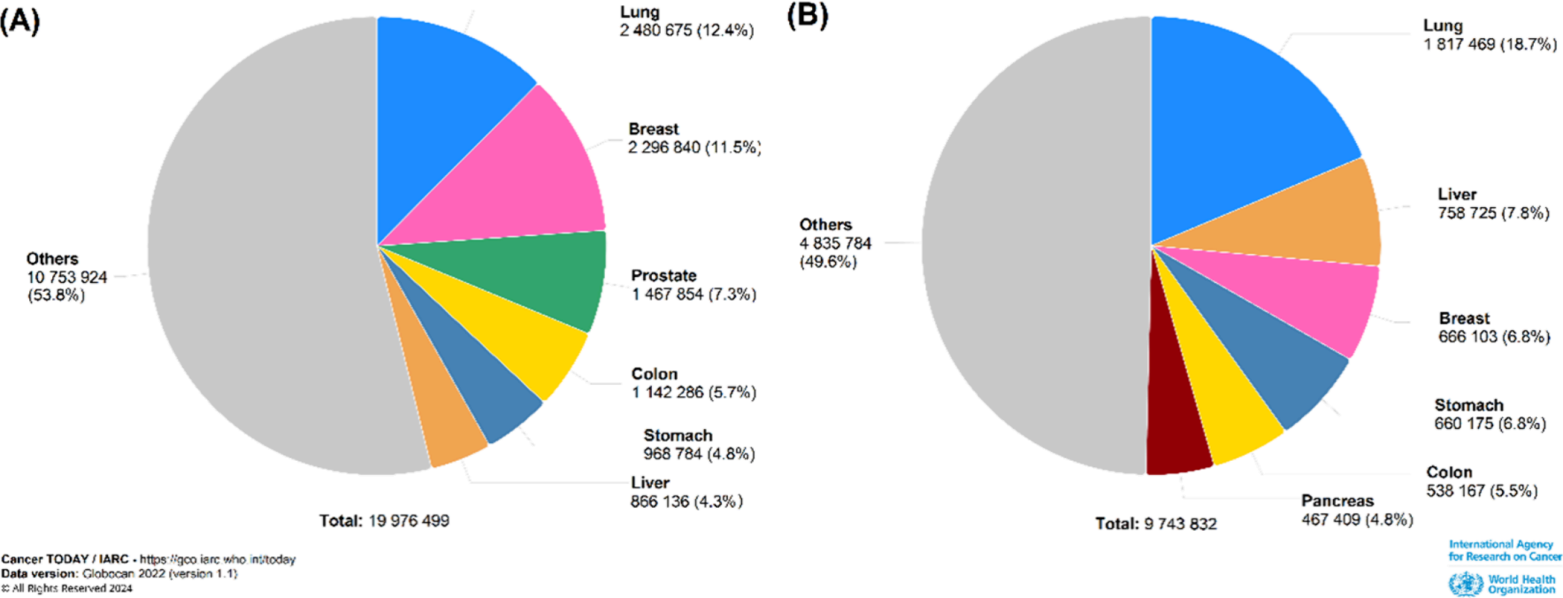
Percentage distribution of cancer incidence (A) and mortality (B) by different types of cancer worldwide according to Globocan 2022. (A) Global total cancer incidence (19,976,499 cases) highlighting breast cancer (11.5%), lung cancer (12.4%), and prostate (7.3%) as the most prevalent types. (B) Global total cancer mortality (9,743,832 deaths) with lung cancer (18.7%) representing the leading cause of cancer-related deaths followed by liver cancer (7.8%). Copyright 2022 Globocan; graphic production: Global Cancer Observatory (http://gco.iarc.fr).

Cancer is the leading cause of death globally, surpassing coronary heart disease and stroke. Studies and clinical trials have been conducted to comprehend and seek a solution to cancer. However, even with advances, cancer remains a significant global health challenge. According to GLOBOCAN 2020, a project of the International Agency for Research on Cancer (IARC), it was estimated that there were over 19.3 million new cancer cases and 10 million deaths in 2020 (Figure 1). These numbers are expected to increase to 22.2 million new cases and 13.2 million deaths in 2030, underscoring the urgent need for effective cancer prevention and treatment strategies.^1−3^

### 1.1 Data Source

Lung cancer is one of the most common and deadly types of cancer, accounting for 18.0% of cancer deaths worldwide. Also known as bronchogenic carcinoma, it can arise in the lung parenchyma or the bronchi. The pathophysiology of lung cancer is extremely complex and not yet fully understood.^4^ Understanding the intricacies of this disease is crucial for developing effective prevention and treatment strategies.

The World Health Organization (WHO) classifies lung cancer into two major histologic categories, according to histological type: (i) small cell lung cancer (SCLC) (∼13% of all cases), characterized by high numbers of mitotic events, genomic instability, high vascularization, and early distant metastasis; and (ii) nonsmall cell lung cancer (NSCLC) (∼85% of all cases), a highly heterogeneous type, subdivided in adenocarcinoma (ADC), squamous cell carcinoma (SCC), and large-cell carcinoma (LCC), which have overall 5-year relative survival rates of 17%, 14%, and 9%, respectively.^5,6^ The most relevant genetic mutations associated with the development of lung cancer occur in the MYC, BCL2, and p53 genes for SCLC, and EGFR, KRAS, and p16 for NSCLC cancer.^4^

The etiology of lung cancer is related mainly to long-term smoking,^3,6^ but there are many other factors, such as environmental exposure due to passive smoking, air pollution, workplace exposures (asbestos, arsenic, ionizing radiation, arsenic, chloromethyl ethers, chromium, isopropyl oil, mustard gas, nickel, beryllium, lead, copper, chloroprene, and vinyl chloride natural radioactive radon gas), as well as the genetic susceptibility, which represents a significant risk factor.^1^

Unfortunately, lung cancer is a silent disease in its early stages (stages I and II) and is usually discovered in more advanced stages (III and IV). The survival rate for patients with lung cancer depends mainly on early diagnosis. When detected in the initial stage, the chances of successful treatment and patient survival can significantly increase.^7^ Currently, conventional imaging techniques (such as X-rays, magnetic resonance imaging, computed tomography, endoscopy, and ultrasound) and tissue morphological analyses (histopathology) or cells (cytology) are used as strategies for early diagnosis. However, imaging methods detect cancer only when there is a visible change in the tissue, which usually occurs when the disease is already in an advanced stage. Moreover, cytology and histopathology are not efficient and accurate in detecting cancer at this initial stage.^8,9^ Diagnosing cancer accurately and assertively is essential to designing better therapeutic strategies and achieving treatment success.

Lung cancer therapy remains a significant challenge for the medical community. Current clinical therapeutic modalities for lung cancer are multidisciplinary, involving surgical resection (such as lung lobectomies and segmentectomies) combined with adjuvant radiotherapy, chemotherapy, immunotherapy, or biological therapy. The choice of treatment depends on the specific type of lung cancer and the overall health of the patient. Despite significant advancements in these techniques, the prognosis and overall survival rate for patients with lung cancer remain low, with a high mortality rate.^5,10^ It is a scenario that draws attention to the urgent need to search for new therapeutic alternatives for this disease.

Nanomedicine has already made significant strides in clinical practice, offering unique opportunities to develop platforms to diagnose, treat, and prevent pulmonary diseases. This promising field of science and technology provides novel and paradigm-shifting solutions to current biomedical problems, particularly in oncology. Conventional treatments for lung cancer pose significant challenges, leading researchers to explore nanotechnology as a promising and effective alternative, offering numerous possibilities for targeted approaches, improving the pharmacokinetic and pharmacodynamic properties of nanoencapsulated drugs while reducing toxicity.^1,5,11,12^ Currently, there are over 50 nanomedicines intended for the treatment of lung cancer,^13^ but only two nanoparticle-based therapies are clinically available: Abraxane (nab-paclitaxel) and Genexol-PM (Cynviloq). Abraxane, the first FDA-approved chemotherapy incorporating albumin into its formulation, is indicated for locally advanced or metastatic NSCLC, while Genexol-PM is approved in South Korea for the treatment of NSCLC.^5,14^

Recently, greater efforts have been devoted to engineering suitable nanotechnological platforms to improve drug delivery to tumor tissues. Despite the advantages of nanotechnology in oncology, one of nanomedicine’s biggest challenges is to overcome the physiological barriers and those imposed by the immune system.^15^ Upon contact with biological fluids, nanoparticles (NPs) can undergo aggregation or be coated by a layer of serum proteins, the so-called “protein corona”, a complex process and structurally variable coating that can substantially affect the stability, biodistribution, and physicochemical properties of the NPs. In this context, knowledge about the disease and the nanosystems’ physicochemical properties is essential to achieve clinical progress. Moreover, the success of treatment using nanotechnology depends on the ability of these nanostructures to evade immune defense cells, cross biological barriers, and accumulate in diseased organs and tissues.^16^

This review aims to highlight the challenges associated with treating lung cancer and provide an overview of recent advancements in nanoplatforms for the early diagnosis and treatment of this disease. Developing effective therapeutic strategies requires a comprehensive understanding of the disease and relevant factors such as clinical outcomes and challenges, physicochemical properties of nanomaterials, nanobio interactions, nanotoxicity, and regulatory considerations to ensure patient safety. By addressing the latter issues, we hope to expand knowledge about lung cancer and demonstrate the potential of nanotechnology as a promising alternative to current treatment methods. The concepts discussed in this review can also apply to other tumor types and diseases within an oncological context and encourage the development of innovative approaches to cancer treatment that can improve patient outcomes and quality of life.

## 2 Etiology and Risk Factors of Lung Cancer

Pulmonary carcinogenesis is a complex and multifactorial process that involves a wide range of contributing factors, including epidemiological, genetic, epigenetic, and nongenetic factors. However, tobacco smoking remains the leading cause of human lung cancer, responsible for approximately 85% of all lung cancer cases. Additionally, smoking contributes to about one-third of all cancer cases globally. Smokers are at a significantly higher risk of developing lung cancer, with a 23-fold increase compared to nonsmokers.^17^

Tobacco smoke is a complex mixture of more than 7,000 potentially carcinogenic substances, most of which are polycyclic aromatic hydrocarbons (PAHs), such as aza-arenes, N-nitrosamines, aromatic amines, heterocyclic aromatic amines, and free radicals, such as hydroquinone and semiquinone quinones, and other harmful components of tobacco smoke.^17,18^ All these substances, upon inhalation, are metabolized by enzymes, such as glutathione S-transferase, uridine-58-diphosphate-glucuronosyltransferase, and sulfatases, and the resulting metabolites can bind to DNA, forming adducts. While some of these adducts are repaired by the DNA repair mechanism, many escape this process and interact with tumor suppressor genes, such as p53 and Kirsten-ras oncogenes (KRAS), leading to carcinogenesis.^17^

**Figure 2:**
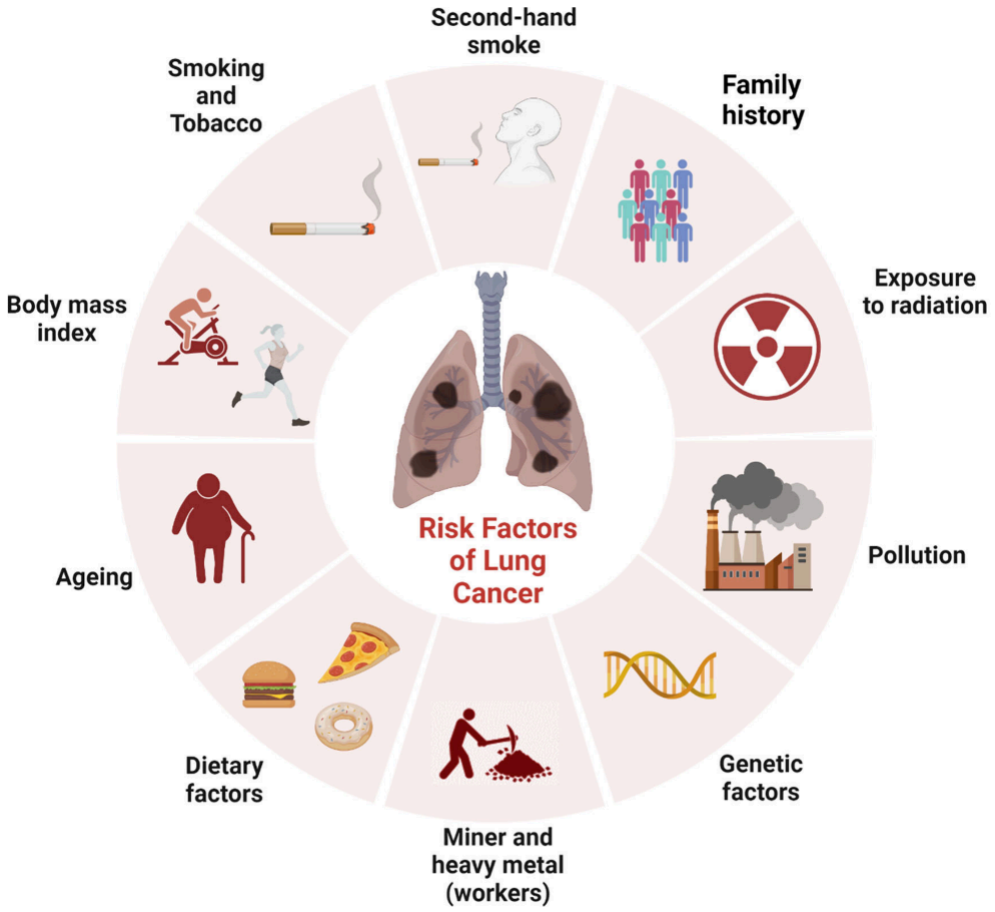
Schematic representation of the various factors related to the development of lung cancer. (Created by authors using BioRender).

Although tobacco smoking is considered the most significant risk factor for lung cancer, it is not the only one. Epidemiological evidence suggests that many cancer cases are related to environmental factors, such as lifestyle, infections, overexposure to sunlight, and chemicals, as well as genetic inheritance (Figure 2). In recent years, there has been an increase in the incidence of lung cancer among nonsmoking individuals, which has been linked to various factors, such as exposure to secondhand smoke and air pollution.^19−22^ Advances in diagnostic technologies have enabled researchers to better understand the molecular mechanisms underlying lung cancer development, leading to the identification of novel risk factors and potential therapeutic targets.

**Figure 3:**
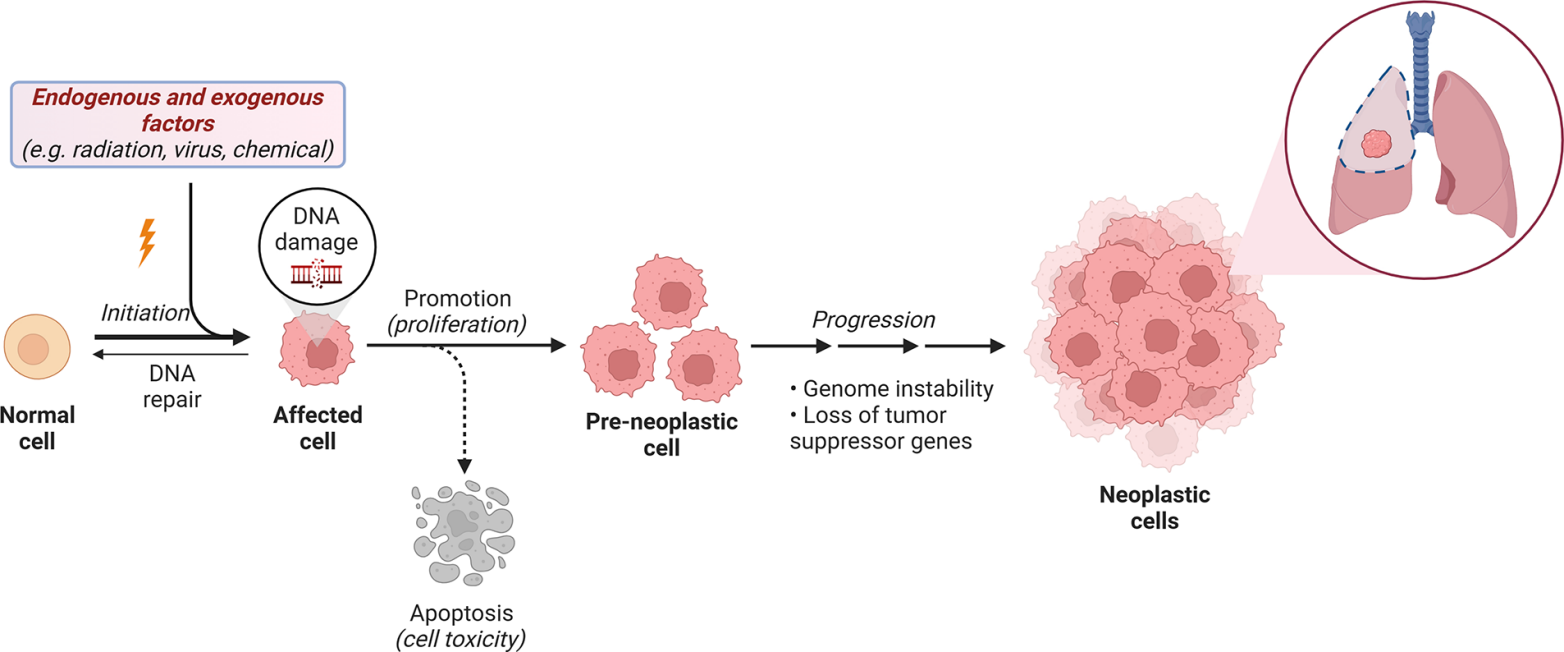
Summary illustration of the carcinogenic process triggered by endogenous and exogenous factors. (Created by authors using BioRender).

Pulmonary carcinogenesis can be triggered by both exogenous and endogenous factors (Figure 3). Exogenous molecules, including chemical substances like epoxides, imines, and aromatic compounds, are considered genotoxic as they can interact and directly damage DNA. Other substances, like chloroform, androgens, and radiation are exogenous nongenotoxic, and induce lung cancer through different mechanisms.^23−25^ Chloroform affects lung tissue through toxic effects on cell membranes, androgens stimulate cancer cell growth via signaling pathways, and low-level radiation exposure can promote lung cancer by damaging lung tissue and promoting cancer cell growth.^24,25^

Endogenous factors arise from normal metabolic processes in the body, producing substances like free oxyradicals, aldehydes, and ketones during respiration or food breakdown by gut bacteria.^23−25^ Exposure to these substances can disrupt cell metabolism, leading to the development of preneoplastic cells that rapidly proliferate due to the loss of tumor suppressor genes and genome instability, ultimately leading to tumor formation (Figure 3).

Occupational exposure to harmful particles and substances in the workplace, as well as factors related to substances emitted by vehicles (polycyclic aromatic hydrocarbons, crystalline silica, heavy metals, arsenic, asbestos, beryllium, cadmium, chloromethyl ethers, chromium, nickel, radon, vinyl chloride, carbon monoxide, ozone, particulate matter, nitrogen dioxide, aldehydes, benzene, 1,3-butadiene, benzopyrene, and metals), play a crucial role in pulmonary carcinogenesis.^26−28^

According to Chen et al. (2014), nitrogen dioxide, a byproduct of vehicle exhaust resulting from the oxidation of nitrogen monoxide, undergoes photochemical reactions that produce substances such as nitrate, sulfate, and organic aerosols. While these substances are known to contribute to air pollution, it is primarily their role in the formation of secondary particulate matter that poses a significant risk to human health. Exposure to these particles has been associated with respiratory issues, oxidative stress, and inflammation, which can indirectly contribute to chromosomal damage, alter cell cycle processes, and affect proteins involved in cell cycle regulation, all of which may influence the development and progression of lung cancer.^29^

## 3 Conventional Treatments and Diagnosis for Lung Cancer

Early diagnosis is crucial for the successful treatme nt of lung cancer. However, the disease is usually asymptomatic in its early stages, making it difficult to obtain an early diagnosis.^30^ Traditionally, standard diagnostic techniques include histological examination of resected tumors and tomography. Imaging technologies such as nuclear magnetic resonance, conventional tomography, positron emission tomography, and single photon emission computed tomography have also provided important anatomical and physiological information about the tumor but require the use of radiolabels or biomolecules labeled to target cells. Biopsy has been sufficient for deciding on the choice of therapeutic intervention, but it presents a potential risk of bleeding in the lung.^31,32^ In this context, nanotechnology represents a promising platform for providing new imaging probes and contributing to early stage diagnosis.^33,34^

Depending on the disease stage at its diagnosis, the conventional therapeutic strategies include chemotherapy, radiotherapy, immunotherapy, and surgery resection.^35^ The latter is the most effective curative modality in the early stages of the disease, however, in metastatic and more advanced stages, the chances of cure are low and are rarely successful. To improve resectability, it is necessary to reduce the size of the tumor, and only after being treated with radiotherapy and/or chemotherapy, the medical team verify the possibility of removing the tumor.^5,36,37^

Radiotherapy is a localized treatment and can be used as curative or palliative treatment across all stages of the disease. Although it has been widely used in the treatment of cancer, mainly associated with chemotherapy, the disadvantage of this therapy is the fact that irradiation can cause skin lesions, as well as pulmonary and cardiovascular alterations. Symptoms of radiation pneumonitis, including low-grade fever, congestion, dry cough, pleuritic chest pain, and a feeling of fullness in the chest, usually develop one to three months after the end of radiation therapy. Another relevant adverse effect is pulmonary fibrosis, which is often permanent and marked by progressive dyspnea.^38^

Chemotherapy is a standard treatment used as neoadjuvant or adjuvant therapy, in combination with radiotherapy. The lack of specificity/selectivity of chemotherapy drugs, unwanted distribution of drugs to healthy organs and tissues, and the predisposition of tumor cells to become more resistant result in the administration of higher doses and, consequently higher toxicity.^39^ Chemoresistance is usually caused by changes that occur within tumor cells, such as overexpression of P-glycoprotein (P-gp), responsible for drug efflux, increased DNA repair activities, cancer stem cell development, and dysregulation of apoptosis in the tumor microenvironment (TME).^40^ The recommended chemotherapy treatment for advanced lung cancer, especially in the case of nonsmall cell lung cancer (NSCLC), involves systemic chemotherapy with platinum derivatives (e.g., cisplatin, oxaliplatin), combined with taxanes (such as Paclitaxel or Docetaxel) or gemcitabine. Other drugs and monoclonal antibodies approved by the FDA are also used as palliative treatments, in combination or not with radiotherapy.^41,42^

Immunotherapy is a newer approach to the treatment of lung cancer that uses the body’s immune system to fight cancer cells. This therapy can be used in combination with chemotherapy, radiotherapy, or as a standalone treatment, depending on the stage and type of lung cancer. Immunotherapy acts by targeting specific molecules on cancer cells or by stimulating the immune system to attack cancer cells more effectively. Some examples of immunotherapy drugs used to treat lung cancer include checkpoint inhibitors, such as Pembrolizumab and Nivolumab, which block signals that prevent the immune system from attacking cancer cells, and monoclonal antibodies, such as durvalumab and atezolizumab, which target specific proteins on cancer cells. Immunotherapy has shown promising results in improving survival rates and reducing the side effects of traditional treatments. However, not all patients are eligible for immunotherapy, and it can cause side effects such as fatigue, rash, and inflammation in some cases.^43^

Tables 1 and 2 provide a comprehensive summary of conventional chemotherapies and immunotherapies for the treatment of lung cancer.

**Table 1:** Currently Approved Chemotherapeutics for the Treatment of Different Types of Lung Cancer

mechanism of action	agent	type of lung cancer	references
DNA cross-linking	Ifosfamide	SCLC and NSCLC	(44,45)
	Platin derivated	Nononcogene addicted advanced NSCLC and advanced nonsquamous NSCLC	(46,47)
	Mitomycin-C	NSCLC	(48,49)
Stabilizes microtubules	Paclitaxel	Advanced and metastatic nonsmall-cell lung cancer (NSCLC)	(50)
	Nab-paclitaxel	Locally advanced nonsmall cell lung cancer or metastasized and relapsed SCLC	(51,52)
	Docetaxel	Advanced and metastatic NSCLC	(53,54)
Inhibits microtubule formation	Vincristine	Small-cell lung cancer	(55)
	Vinblastine	NSCLC	(56,57)
	Vinorelbine	Advanced NSCLC	(58,59)
Topoisomerase I inhibitor	Topotecan	Patients with relapsed SCLC	(60)
	Irinotecan	SCLC and recurrent SCLC	(61,62)
Topoisomerase II inhibitor	Etoposide	Patients with extensive (metastatic) small-cell lung cancer	(63,64)
	Doxorubicin	Small-cell lung cancer	(65)
DNA alkylating agent	Cyclophosphamide	SCLC and NSCLC	(66)
	Temozolomide	Previously treated SCLC	(67,68)
	Mechlorethamine	SCLC	(69)
Folate antimetabolite	Pemetrexed	SCLC and NSCLC	(70−72)
Folate antimetabolite	Methotrexate	NSCLC	(73)
Nucleoside analogue	Gemcitabine	Early and advanced NSCLC relapsed SCLC.	(74−76)

**Table 2:** Currently Approved Small Molecules and Immunotherapeutic Agents for the Treatment of Different Types of Lung Cancer

mechanism of action	agent	type of lung cancer	references
EGFR inhibitor	Erlotinib	Advanced EGFR mutation-positive NSCLC	(77,78)
	Gefitinib	Advanced NSCLC patients harboring activating EGFR mutations	(79,80)
	Cetuximab	Advanced NSCLC	(81)
	Osimertinib	NSCLC harboring a common EGFR mutation	(82,83)
	Necitumumab	Advanced squamous nonsmall-cell lung cancer	(84,85)
	Dacomitinib	Patients with advanced EGFR mutation-positive nonsmall-cell lung cancer	(86,87)
EGFR/HER2 inhibitor	Afatinib	First-line therapy of *EGFR* mutant NSCLC patients and metastatic NSCLC whose tumors have nonresistant *EGFR*	(88−90)
ALK/ROS1 inhibitor	Crizotinib	Advanced ALK-positive NSCLC and patients with ROS1-rearranged advanced nonsmall-cell lung cancer (NSCLC)	(14,91,92)
	Lorlatinib	Patients with ALK-positive advanced NSCLC and ROS1-positive advanced/metastatic NSCLC	(62,92)
ALK inhibitor	Ceritinib	Advanced ALK-rearranged NSCLC	(93,94)
	Alectinib	Patients with ALK-positive advanced/metastatic NSCLC	(95,96)
	Brigatinib	Patients with ALK-positive advanced/metastatic NSCLC	(97,98)
VEGF-A inhibitor	Bevacizumab	Patients with unresectable or metastatic nonsquamous NSCLC	(99,100)
	Ramucirumab	Patients with NSCLC with disease progression or after platinum-based chemotherapy	(101,102)
VEGFR/FGFR/PDGFR inhibitor	Nintedanib	Advanced NSCLC	(103,104)
mTOR inhibitor	Everolimus	Advanced NSCLC	(105,106)
BRAF inhibitor	Dabrafenib	NSCLC harboring BRAF mutation	(107,108)
MEK inhibitor	Trametinib	NSCLC harboring KRAS mutation	(109,110)
PD-1 inhibitor	Nivolumab	Advanced squamous and nonsquamous NSCLC with progression or after platinum-based chemotherapy and patients with recurrent SCLC	(111−113)
	Pembrolizumab	Advanced squamous and nonsquamous NSCLC with progression or after platinum-based chemotherapy	(112,114,115)
PD-L1 inhibitor	Atezolizumab	Metastatic nonsquamous NSCLC and PD-L1-positive NSCLC that progressed during or after standard treatments	(112,116,117)
	Durvalumab	Locally advanced NSCLC	(112,118)

## 4 Physicochemical Properties of Nanocarriers and Their Application in the Diagnosis and Treatment of Lung Cancer

Nanotechnology has opened new avenues for the diagnosis and treatment of cancer. Lung cancer is a complex and challenging disease to manage, with limited treatment options. However, recent advances in nanotechnology have led to the development of innovative nanomaterials that can specifically target cancer cells while minimizing harm to healthy tissues. In this section, we will delve into the physicochemical properties of nanomaterials and discuss their potential benefits for the diagnosis and treatment of lung cancer.

**Figure 4:**
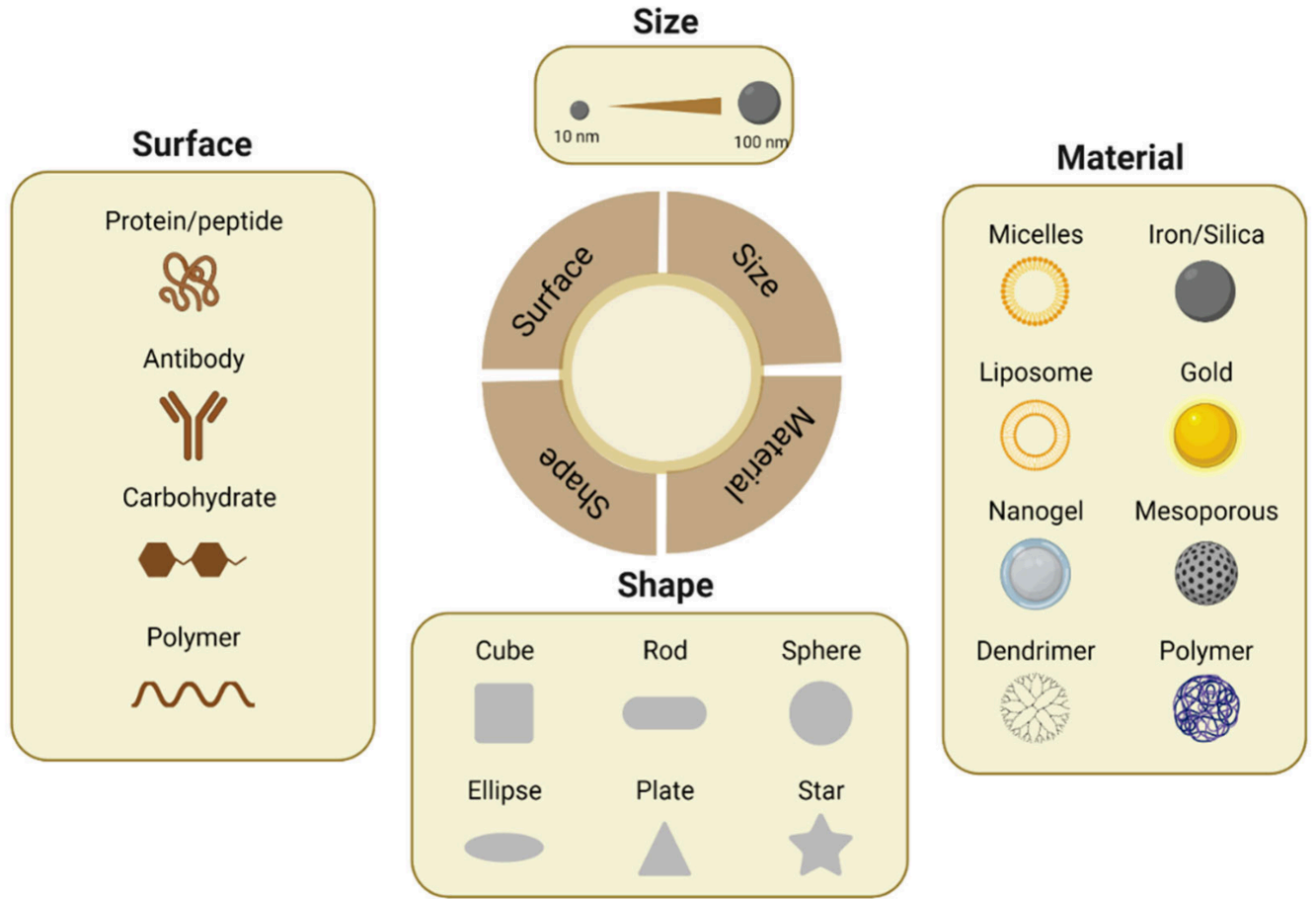
Summary of different properties and characteristics that make it possible to work with NPs, including size, materials, shape, and surface. (Created by authors using BioRender).

The manipulation of the physicochemical properties of nanosystems by their intended application is a key feature of nanotechnology. The material, size, shape, and surface characteristics of NPs are among the most significant properties that impact their performance and biodistribution in the biological environment^119,120^ (Figure 4). These features enable nanosystems to overcome the physiological barriers, besides protecting drugs from early inactivation or biodegradation, overcoming multidrug resistance and efflux transporters, prolonging circulation time, enhancing lipophilicity, and promoting permeability through biological barriers.^1,121,122^

All the physicochemical aspects highlighted in Figure 4 must be considered for the effective design of nanosystems for medical applications. The appropriate nanoparticle **size** is crucial for drug accumulation at the tumor site, cellular uptake, release profile, stability, targeting, biodistribution, tumor penetration, and circulation half-life. Smaller NPs can easily permeate through biological barriers and have been shown to induce cellular apoptosis. However, the “ideal” size range depends on the pathology and route of administration. For instance, particles smaller than 10 nm are rapidly eliminated by renal and hepatic clearance, whereas those larger than 100 nm are phagocytized by alveolar macrophages.^42,123^

Selecting the “ideal” size also requires careful consideration of various factors, including physiological barriers, biodistribution, and clearance, among others.^129^ Systems with sizes ranging from 10 to 100 nm have maximum cellular uptake in nonphagocytic cells, while those with a diameter of 40–50 nm have higher cell internalization efficiency.^129,130^ NPs larger than 200 nm activate the complement system and accumulate in the liver and spleen. Additionally, size is an important parameter when the objective is targeting pulmonary tumor vasculature, where pores are typically 40–200 nm in diameter.^124,125^

Particle size is an important factor for phagocytosis, mainly occurring in the deep lungs, where dendritic cells, neutrophils, and alveolar macrophages encounter inhaled particulate matter. Micrometer-scale particles are more easily detected and phagocytized by alveolar macrophages than nanoscale structures.^126^ Alveolar macrophages play a crucial role in internalizing and digesting xenobiotics and can phagocytize particles with sizes between 0.25 and 3 μm. On the other hand, smaller particles (≤0.25 μm) are phagocytized by dendritic cells. Therefore, when designing drug delivery systems intended for pulmonary administration, particle size must be taken into consideration. Particles with sizes between 0.25 and 3 μm are more likely to be internalized by alveolar macrophages, while particles ≤0.25 μm can be internalized by dendritic cells.^127^

Engineered gold NPs of different sizes were utilized to investigate the impact of particle diameter on tumor xenograft targeting. The results showed that actively targeted NPs within the 60 nm diameter range had higher accumulation rates in tumors than their passive counterparts.^128^

In another study, Kulkarni et al.^129^ focused on investigating the effects of particle size and surface coating on the cellular uptake of polymeric NPs coated with d-alpha-tocopheryl polyethylene glycol 1000 succinate (TPGS) for drug delivery across physiological barriers, such as the gastrointestinal (GI) barrier for oral chemotherapy and the blood-brain barrier (BBB) for brain cancer imaging and therapy. The *in vivo* studies revealed that particle size and surface coating significantly influenced the biodistribution of systems after intravenous administration. TPGS-coated NPs of smaller size (<200 nm) escaped recognition by the reticuloendothelial system (RES), prolonging the NPs’ half-life in the blood system. TPGS-coated NPs of 100 and 200 nm also showed potential for drug delivery across the GI barrier and the BBB.^129^

Nanoparticle **shape** also is an important parameter that can affect cellular uptake, blood circulation, antitumor activity, and biodistribution. By modifying the shape of NPs, it is possible to enhance their accumulation in the tumor microenvironment. NPs can be synthesized in different morphologies, including nanotubes, nanofibers, nanospheres, or nanoflowers (Figure 4). Additionally, 2D structures such as nanoplates or nanosheets offer unique advantages, such as larger surface areas for drug loading and enhanced interaction with biological environments. These flat, sheet-like structures can influence cellular uptake and biodistribution in ways that differ from more conventional nanoparticle shapes, making them a promising option for specific biomedical applications.^130^

Characterizing the shape of NPs is critical for understanding their interactions with biological systems. Evaluating the contact angle formed after interaction with macrophages can help determine the rate of internalization and phagocytosis by these cells. For spherical NPs, their internalization rate is independent of the contact angle due to their symmetrical shape.^131^ However, nonspherical NPs may have less physicochemical stability than spherical ones, which can be overcome with optimized production methods or materials modifications. It is also essential to note that nonspherical shapes may not present a homogeneous charge distribution, and more sensitive methods than dynamic light scattering may be required for accurate measurements.^132^

Kaplan, M. et al.^132^ evaluated the delivery dynamics of spherical, rod, and elliptical disk PLGA NPs on lung cancer, *in vitro* and *in vivo*. Spherical shape NPs released 80% more drug than the rod and disk-shaped ones, due to the different surface area and porosity. The uptake and intracellular traffic were faster for spherical NPs, followed by the nanorods and nanodisks, as demonstrated by Li et al.^133^ in their theoretical study.

The **surface charge** of NPs can affect their stability, bioavailability, and interaction with biological substrates. Zeta potential is commonly used to measure the surface charge of nanosystems, with high values indicating a greater degree of ionization and improved electrostatic repulsion between particles. The latter strategy reduces aggregation and improves the electrical stability of the system, as suggested by Parveen and Sahoo.^134^

The surface charge also plays a critical role in the biological performance of nanosystems, especially in the tumor microenvironment, where most biomolecules and biological substrates are ionized. Positively charged NPs can easily penetrate tumor cell membranes due to the electrostatic interactions with negatively charged tumor membranes. On the other hand, negatively charged NPs can interact with biological substrates through various supramolecular interactions, including hydrogen, ionic, covalent, van der Waals, and hydrophobic interactions.^42^

NPs with a surface charge close to zero interact less with the endoplasmic reticulum and remain longer in the bloodstream. As a result, they are considered a more suitable choice for interaction and penetration into cellular structures.^131,135^ Given the importance of surface charge in ensuring the physical-chemical stability of nanosystems and their interaction with biological substrates, nanotechnology allows for the modulation of these properties to achieve the intended application’s purpose.

**Figure 5:**
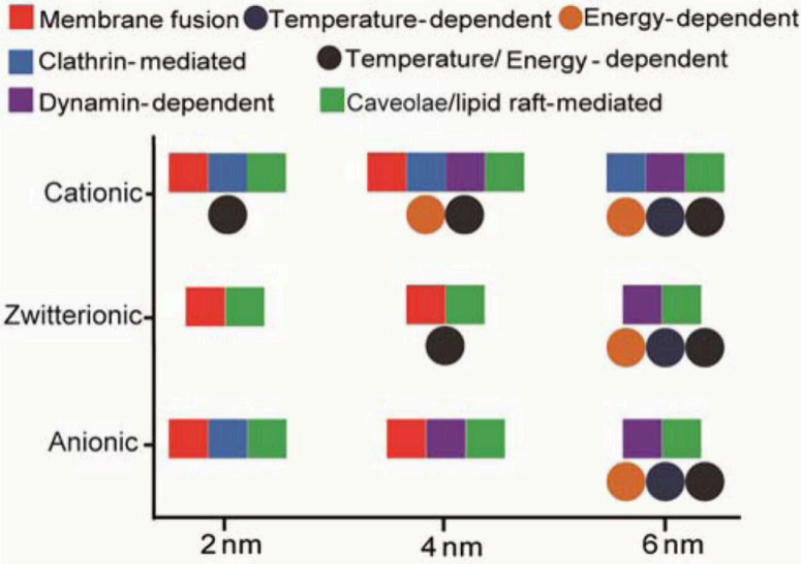
Interplay of size and surface functionality on the cellular uptake pathway of gold NPs. Reproduced with permission from ref (145). Copyright 2015 American Chemical Society.

The charge on the surface of NPs has a significant impact on their interaction with cell membranes and subsequent cellular uptake. The surface charge of cell membranes is negatively charged. In this case, cationic-charged NPs have a stronger interaction with the phospholipid membrane of cells than anionic and neutral ones, allowing them to adhere readily to the cell membrane and increase the membrane-engulfing process. On the other hand, negatively charged NPs induce local disorders in the cell membrane that make their interaction unfavorable.^136^ Cho et al.^137^ showed that cationic gold NPs have a 5-fold greater uptake compared to their anionic counterparts, and can even directly diffuse into cells by generating holes in the cell membrane. In another study by Arvizo et al.^138^ cationic gold NPs caused a depolarization of the cell membrane, leading to reduced viability and proliferation of normal cells by changing intracellular pathways. Hauck and colleagues^139^ investigated the uptake of highly positive and highly negative gold nanorods in HeLa cells at varying concentrations and found that maximum uptake occurred with positively charged NPs, while minimum uptake occurred with negative NPs. In a study by Jiang and colleagues,^140^ it was shown that surface charge can affect the size-dependent uptake of NPs in cells. Anionic gold NPs showed a decrease in cellular uptake as their size increased, while cationic gold NPs exhibited an increase in internalization with decreasing size (Figure 5).

The surface property of NPs, as **hydrophilic/hydrophobic** characteristics, also plays an important role in their ability to interact with biological substrates. The high hydrophobicity of NPs favors cell uptake^141,142^ due to the recognition of the hydrophobic surface of cell membranes lipid tails (see Figure 5), besides contributing to higher adsorption of plasma proteins, allowing them to be recognized and captured by endoplasmatic reticulum, undergoing faster blood clearance. In some studies, the functionalization of the nanocarriers with hydrophilic ligands and polymers (for example, poly(ethylene glycol) [PEG], poloxamer, dextran, chitosan, poloxamine, pluronic F127, poly(oxyethylene)) has been described as a rational strategy to reduce rapid clearance and extend circulation time. Furthermore, the hydrophilicity/hydrophobicity balance of amphiphilic polymers is a key factor in their self-assembly. Stimuli, such as temperature and pH can destabilize the structure and promote drug release.^119^

The **mucoadhesive capacity** of nanostructured systems is a surface characteristic that is also important when the goal is to prolong contact with mucosal surfaces. In general, mucosal surfaces are negatively charged due to sialic acid residues from mucin (MUC) chains. MUC, an extremely heterogeneous glycoprotein, has alternating hydrophobic and hydrophilic regions. The interaction of MUC with nanomaterials is driven by interactions between charges of residues of the protein backbone, such as the aspartic and glutamic acid residues (p*K*_a_ ∼ 4), and the charges of side chain oligosaccharides such as sialic acid residues (p*K*_a_ ∼ 2.6) and sulfate groups (p*K*_a_ ∼ 1). The properties of MUC are controlled by complex interactions between electrostatic repulsive forces and associative interactions of hydrophobic microdomains, mainly in nonglycosylated regions rich in cysteine.^143,144^ A mucociliary clearance occurs through two processes: (i) filtering by size, in which the mucus can retain particles above 100 nm; (ii) and reflective filtration, where particles smaller than 100 nm are retained due to covalent or supramolecular interactions (hydrogen bonding, electrostatic and hydrophobic reflections, and other specific protections).^145−147^

Dyawanapelly et al.^148^ showed that the surface modification of polymer NPs with chitosan and chitosan oligosaccharides significantly improved mucoadhesion and cell uptake of proteins. These NPs were found to be stable and safe, with no significant toxicity observed *in vitro*. Similarly, Butnarasu et al.^149^ reported the development of mucosomes, which are intrinsically mucoadhesive glycosylated mucin NPs, as a multidrug delivery platform. These NPs were found to efficiently bind to mucosal surfaces, improving the retention time and enhancing the uptake of drugs by target cells. Moreover, these mucosomes were shown to be biocompatible and capable of delivering multiple drugs simultaneously. Overall, the studies demonstrate the potential of mucoadhesive NPs as an effective drug delivery platform for mucosal surfaces, with improved retention time, cell uptake, and biocompatibility.

Several studies have shown that the mucoadhesive properties of NPs can improve their interaction and internalization in lung cancer cells. Shin et al.^150^ reported that mucoadhesive NPs loaded with paclitaxel showed increased cellular uptake and improved anticancer efficacy in several cancer types, including lung cancer cells. In another study, Alhakamy and Md^151^ developed chitosan-coated PLGA NPs loaded with itraconazole (ITR) as a potential treatment for nonsmall cell lung cancers. The NPs’ mucoadhesive properties coming from the chitosan coating contributed to transport into the cell, favoring the internalization of the drug and overcoming limitations imposed by the efflux mediated by P-gp.

In conclusion, nanotechnology has opened new possibilities for the diagnosis and treatment of complex diseases like lung cancer. Physicochemical properties such as size, shape, and surface characteristics play a crucial role in the performance of nanomaterials in the biological environment. When designing drug delivery systems intended for pulmonary administration, particle size must be taken into consideration, as it can affect drug accumulation at the tumor site, cellular uptake, release profile, stability, targeting ability, biodistribution, tumor penetration, and circulation half-life. Nanoparticle shape is also important, as it can affect cellular uptake, blood circulation, antitumor activity, and biodistribution. By manipulating these properties, it is possible to develop innovative nanomaterials that can specifically target cancer cells while minimizing harm to healthy tissues. The use of nanotechnology in the diagnosis and treatment of lung cancer holds great promise and may revolutionize the field of oncology in the future.

## 5 Physiological Barriers and Nanotechnology Solutions for Pulmonary Drug Delivery

The treatment of lung cancer is often complicated by multiple factors, including difficulties in early diagnosis, intra- and intertumoral heterogeneity, tumor biology, and the ineffectiveness of current treatments. The tumor microenvironment (TME) also imposes significant challenges due to its complexity and heterogeneity, with extracellular matrix and vasculature playing key roles in limiting the success of cancer treatment.^5^ To effectively treat lung cancer, drugs need to be delivered to the lungs in a manner that specifically targets neoplastic cells while avoiding harm to normal cells.

Traditional chemotherapy for lung cancer is commonly administered orally or intravenously. These routes of administration often lead to systemic side effects, such as nausea, diarrhea, and gastrotoxicity, because of antineoplastic agents. Although inhalation chemotherapy offers the advantage of site-specific delivery and direct contact of the drug with the pulmonary mucosa, its effectiveness is still hampered by local pulmonary barriers. Specifically, physiological barriers like the pulmonary air-blood barrier, mucociliary clearance, mucus, pulmonary surfactant, and macrophage-mediated clearance pose significant challenges to the effective delivery of drugs to the lungs, thereby reducing the overall efficacy of inhaled chemotherapy.^152^

Nanotechnology represents an important strategy to overcome the physiological barriers that hinder the effective delivery of drugs to the lungs. Nanocarriers specific properties such as size, morphology, hydrophobicity, and surface chemistry, allow to enhance drug delivery to the lungs and improve treatment outcomes enabling the targeted delivery of drugs specifically to neoplastic cells.

The following sections will explore how NPs can be designed to improve drug delivery and efficacy by navigating the complex pulmonary environment and minimizing barriers to treatment.

### 5.1 Advantages of Nanoparticles in Overcoming Pulmonary Barriers

#### 5.1.1 Deep Penetration into Lung Tissue

Nanocarriers intended for the treatment of pulmonary diseases, especially cancer, can be administered mainly via inhalation and intravenous routes. In the case of intravenous administration, it should be considered that the systems can be excreted prematurely due to first-pass metabolism. Additionally, studies^153,154^ show that administered nanomaterials can extravasate through the vasculature due to the Enhanced Permeability and Retention (EPR) effect, which is common in tumors. This approach allows passive drug targeting; however, drug delivery for this approach is limited due to the vasculature heterogeneity caused by the EPR effect and the slow leakage rate through the vessels. This can result in the excretion or early metabolism of drugs before they reach therapeutic levels in the tumor.^155^

One way to overcome this limitation and increase the permeability of the tumor vascular system is to utilize chemical and pharmacological substances that promote the dilation of the microcirculation of vessels. The use of these substances will lead to increased blood flow and vascular permeability, ensuring the accumulation of nanocarriers at the target site and enhancing the efficacy of nanosystems. Examples of these dilatation promotors include vascular normalizers such as antiangiogenics (mAbs), which improve blood flow; cotherapy with fibrinolytic drugs that help dissolve fibrinous in occluded vessels; and vascular mediators like bradykinin B1 and B2 activators to enhance vasodilation or angiotensin-converting enzyme inhibitors.^156^ The use of these vascularization promoters, in conjunction with nanocarriers, leads to an increased local concentration of the drug in the TME, thereby ensuring treatment efficacy.

Another option would be physical approaches, which involve strategies such as hyperthermia, photodynamic activation of tissues, radiotherapy, and ultrasound. The effort to study the EPR effect combined with nanotechnology is worthwhile, as some EPR enhancement strategies developed based on nanomedicine are currently in clinical trials (NCT01847326, NCT03107182, NCT00404404, among others.^156^

**Figure 6:**
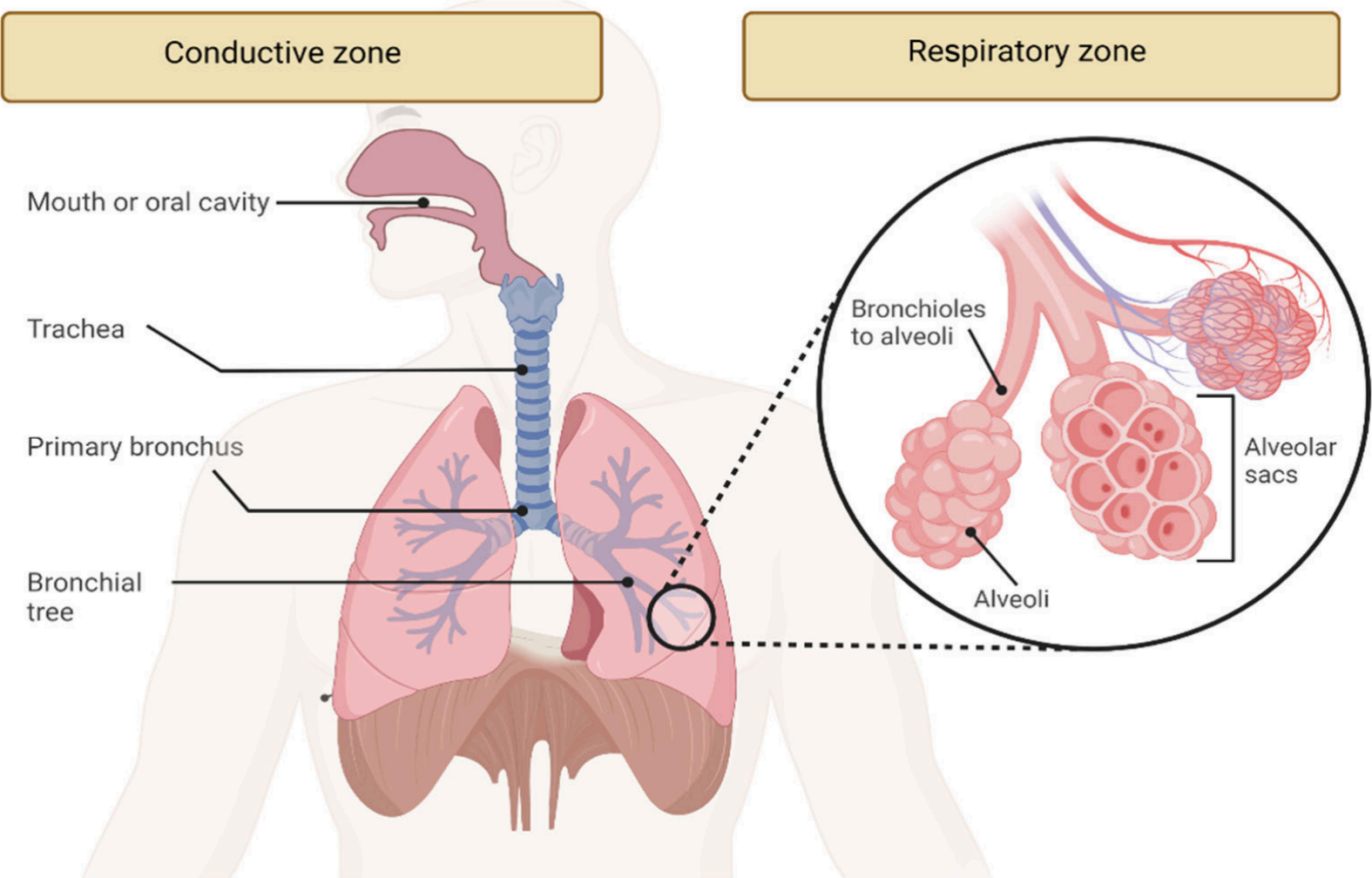
Zone of the respiratory tract (Created by authors using BioRender).

Inhalatory pulmonary drug delivery is a highly promising approach for the treatment of lung diseases. The respiratory tract is a complex system of airways that can be categorized into two main zones: the conducting airways and the respiratory zones,^117,118^ as shown in Figure 6. Each region of the respiratory tract has unique physical and chemical barriers that protect the lungs from harmful agents and impose limitations on drug release.

As the drug travels through the conducting zone, it can either be expelled from the lungs or deposited on the walls of the respiratory tract. Specifically, this particle deposition in the respiratory tract can occur through three main mechanisms: inertial impaction (for particles larger than 10 μm), gravitational sedimentation (for particles between 0.5 and 2 μm), and diffusion (for particles smaller than 0.5 μm, which can be exhaled). In this context, properties such as particle size, morphology, geometry, and surface characteristics play crucial roles in the deposition process and can be modulated to avoid deposition along the conducting zone, allowing the systems to reach the site of action.^157^

Although inhalatory pulmonary drug delivery provides the distinct advantage of bypassing first-pass metabolism, leading to higher bioavailability, delivering the drug directly to the lungs through nanocarriers ensures more precise targeting of pulmonary tumors,^155^ it should be considered that the fate of inhaled nanomaterials is largely determined by their distribution within the lungs, as the deposition is a complex function influenced by absorption kinetics and nonabsorptive clearance mechanisms. Once deposited, nanomaterials come into contact with the mucosal layer and pulmonary surfactant within the airways, which represent one of the most important barriers in the lung.^158^

#### 5.1.2 Surpassing the Pulmonary Surfactant Barrier

Pulmonary surfactant is one of the most important barriers in the lung, responsible for protecting the alveoli from collapsing during breathing and preventing particles from passing through, mainly when administered via inhalation. The surfactant is composed of 90% lipids and 10% specific alveolar proteins and covers the alveoli and ramifications in the peripheral portion of the lung. Once inhaled particles overcome the mucosal barrier, they undergo inevitable adsorption of lipids and proteins on their surface, leading to the formation of the “protein corona”.^159,160^

Despite the challenges posed by the pulmonary surfactant as a barrier, it can also serve as a gateway for innovative treatments. In therapeutic applications, exogenous pulmonary surfactants are utilized to manage various respiratory conditions. These surfactants can replace or supplement the natural (endogenous) surfactant that may be absent or insufficient due to certain diseases. When endogenous surfactant is compromised, exogenous surfactant can function effectively, providing intrinsic prophylactic and therapeutic benefits, either alone or in conjunction with drugs or nanosystems.^161^

To optimize drug delivery systems for the lungs, it is essential to understand the physicochemical properties of NPs and their interactions with pulmonary surfactants. The size, chemical nature, hydrophobicity, and charge of nanocarriers can influence the surfactant in diverse ways, ranging from extracting specific molecules and forming a corona to causing structural reorganization. These functional consequences need careful analysis to avoid toxicological effects and achieve synergistic effects.^161^

When NPs enter the biological environment, such as the pulmonary system, they quickly interact with various biomolecules, including proteins, lipids, and other macromolecules. These biomolecules adsorb onto the surface of the NPs, forming the complex protein corona. This formation is a significant phenomenon because it substantially alters the physicochemical properties of the NPs, such as their size, surface charge, and hydrophobicity. Consequently, this affects how NPs interact with cells, tissues, and the immune system. The protein corona can influence the biological identity of the NPs, affecting their biodistribution, cellular uptake, and clearance from the body. Moreover, the composition of the protein corona is dynamic and can change as NPs move through different biological compartments. Understanding and controlling the formation of the protein corona is essential for optimizing drug delivery nanosystems, as it can enhance targeting specificity and therapeutic efficacy while minimizing potential adverse effects.^162^

A very interesting strategy that has been widely explored is the NPs coating with surfactant components. For example, dextran-based nanogels coated with surfactant components showed high efficacy in delivering siRNA to lung epithelial cells and macrophages, suggesting enhanced distribution and internalization of the NPs and their cargo.^163,164^ Additionally, NPs containing surfactant components have demonstrated their ability to efficiently deliver materials for correcting genetic deficiencies in vivo using emerging gene-editing technologies.^165^ This innovative approach paves the way for groundbreaking respiratory therapies by leveraging the properties of surfactant-coated nanomaterials.

One strategy to mitigate the effects of the protein corona involves preparing or coating NPs with polymeric materials that repel proteins. Functionalization with zwitterionic ligands such as cysteine, targeting molecules like biotin, or hydrophilic polymers like polyethylene glycol (PEG) can help reduce protein corona formation. The effectiveness of these coatings depends on factors such as their density, size, and heterogeneity. Additionally, other approaches include conjugating NPs with antibodies or coating them with fractions of the protein corona to ’camouflage’ the particles, thereby minimizing further protein adsorption.^166^

Coating NPs with hydrophilic polymers like PEG creates a hydrated layer on the NP surface, reducing opsonization and clearance by macrophages. This functionalization enhances NPs stability in biological environments, allowing for controlled and sustained therapeutic release. Incorporating stimuli-responsive materials into NPs that react to tumor microenvironment triggers (e.g., pH, temperature, enzymes) provides targeted drug delivery, minimizing off-target effects.^167^

#### 5.1.3 Overcoming the Mucus Barrier

The mucus is a significant physiological barrier located in the central region of the lung. Once deposited in the respiratory tract lining, nanocarriers come into contact with the mucosal layer. The mucus, secreted by goblet cells and submucosal glands, forms a dense viscoelastic hydrogel that is 5–55 μm thick, rich in electrolytes, proteins, and glycoproteins (mucins), covering epithelial cells. This composition can vary depending on the pathological condition.^146,168,169^ The primary function of mucus is to promote the clearance of inhaled particles, bacteria, toxins, allergens, and other substances, preventing them from reaching the epithelium. While gaseous substances, ions, nutrients, and proteins can easily diffuse through the mucus, particulate substances are immobilized and removed before they can reach the underlying epithelial cells.^144,169^ Depending on their size and physicochemical properties, inhaled particles can be eliminated through three primary mechanisms: mucociliary clearance, phagocytosis, and systemic uptake.^170^

Mucociliary clearance also represents one of the most important defense mechanisms for removing inhaled particles. The cilia or periciliary layer is located just below the mucus layer. This layer is composed of mucin, with monomers connected via cysteine bridges, forming mucin fiber networks that confer viscosity to the mucus. Inhaled particles are easily trapped by the viscoelastic mucus and the periciliary layer, causing the cilia to move rhythmically, pushing the particles along with the mucus toward the pharynx for expulsion. In this context, the size and surface characteristics of particles can be modulated to increase their mucus-penetrating ability. Surface charge, for example, is a critical factor; negatively charged particles tend to penetrate more easily than positively charged ones, which interact with negatively charged mucin and may remain longer in the mucosal layer, increasing the chance of being cleared by mucociliary action.^152,155^

Nanoparticle-based drug delivery has shown significant promise in overcoming the mucus barrier. Studies have demonstrated that smaller particles, particularly those under 300 nm, can effectively penetrate mucus and deliver drugs to epithelial cells. Moreover, surface modifications to NPs, such as incorporating hydrophilic polymers like polyethylene glycol (PEG), have proven effective in reducing adhesive interactions with mucus, thereby enhancing penetration and drug delivery efficiency.^169^ The use of mucoadhesive polysaccharides such as chitosan (CS) further augments this approach. Derived from chitin, chitosan offers biocompatibility, biodegradability, and adaptability, making it superior to other polymers for biomedical applications. Its mucoadhesive properties and ability to reversibly open tight junctions enhance drug absorption and regulate drug release. However, its application in pulmonary delivery is limited by its tendency to aggregate and reduce surface charge at physiological pH, necessitating chemical modifications to improve solubility.^171^

Other natural polymers like hyaluronic acid (HA) and synthetic polymers like poly(lactic-*co*-glycolic acid) (PLGA) have been explored for their potential to enhance drug delivery efficiency and prolong drug release in the lungs. HA’s bioadhesion and role in inflammatory mediation, along with PLGA’s biocompatibility and adjustable degradation rates, make these polymers valuable for advanced pulmonary drug delivery systems.^171^

#### 5.1.4 Overcoming Protein/Efflux Transporter Barriers

After traversing the physical barriers of mucus and intercellular tight junctions, drugs or NPs must overcome chemical barriers related to uptake mediated by proteins and efflux receptors, such as glycoprotein P (P-gp) and metabolizing enzymes present in epithelial cell membranes. P-gp is a transmembrane transporter protein responsible for the efflux of drugs and other molecules from the cell. This protein is overexpressed in tumor cells and is one of the main reasons for multidrug resistance (MDR) in cancer chemotherapy. Most first-line antineoplastic agents used in lung cancer chemotherapy, such as paclitaxel, cisplatin, and doxorubicin, are substrates for P-gp, which decreases treatment efficacy and creates multidrug resistance.^172,173^

By overcoming P-gp-induced efflux, nanocarriers facilitate the delivery of drugs to the target cells. Nanotechnology offers innovative solutions to overcome the MDR, mainly by inhibiting the P-gp efflux mechanism. This inhibition results in increased drug accumulation in tumor cells and potentially enhances cytotoxicity. Nanocarriers also can avoid the efflux pump expressed on the cell membrane by entering the cell through endocytosis or phagocytosis. This ability to bypass the efflux system allows nanocarriers to deliver drugs directly to target cells more effectively.^172^

Various types of nanocarriers, including liposomes, metallic NPs, solid lipid nanocarriers, dendrimers, nanogels, micelles, and polymeric carrier systems, have demonstrated synergistic effects in eliminating cancer cells by inhibiting P-gp. The use of these nanomaterials or combinations of materials, such as TPGS, PEG–PLGA, soluplus, and poloxamer, is widely employed to control drug resistance caused by P-gp efflux.^174−176^

The combination of a P-gp inhibitor with a chemotherapeutic agent within a single nanocarrier further enhances the therapeutic efficacy of anticancer drugs. Besides P-gp inhibitors, anticancer drugs with P-gp inhibitory pharmaceutical excipients or nanomaterials offer significant advantages in controlling drug resistance caused by P-gp efflux. Some nanocarriers themselves possess P-gp inhibitory properties and have been extensively applied in drug-delivery systems to combat P-gp-mediated MDR. However, it is important to note that these inhibitory nanomaterials can also alter the pharmacokinetic profile of coadministered drugs, similar to conventional P-gp inhibitors.^172^

Strategics use of nanotechnology in overcoming P-gp-mediated MDR presents a promising avenue for enhancing the efficacy of lung cancer treatments. By enabling more effective drug delivery and accumulation in tumor cells, nanocarriers play a critical role in improving therapeutic outcomes and advancing cancer care.

Overcoming the challenges of pulmonary drug delivery for lung cancer treatment necessitates addressing complex physiological barriers and the tumor microenvironment. While traditional methods like oral and intravenous administration often result in systemic side effects and limited tumor targeting, inhalation chemotherapy offers direct contact with the pulmonary mucosa but is still impeded by local barriers such as the pulmonary air-blood barrier, mucociliary clearance, mucus, and macrophage-mediated clearance.

Nanotechnology provides a promising solution through the development of advanced nanocarriers designed to effectively navigate these barriers. Tailoring nanoparticle (NP) properties—such as size, morphology, and surface chemistry—can significantly enhance drug delivery precision. Strategies like coating NPs with hydrophilic polymers or surfactant components, enhancing vascular permeability with chemical agents, and employing physical methods like hyperthermia and ultrasound are proving effective in improving delivery outcomes.

Further innovations include overcoming mucus barriers with smaller, surface-modified particles and utilizing natural and synthetic polymers to enhance drug delivery. Addressing challenges like the protein corona and efflux transporters highlights nanotechnology’s potential to address multidrug resistance and optimize therapeutic efficacy.

In summary, leveraging nanotechnology to tackle the multifaceted barriers of pulmonary drug delivery holds substantial promise for enhancing targeting specificity and improving treatment outcomes for lung cancer. Continued exploration and development of these nanotechnology-based solutions are essential for advancing personalized and effective treatment strategies.

## 6 Nanotechnology Approaches for Early Diagnoses of Lung Cancer

It is evident that the success of cancer lung therapies depends on their detection at the early stages, and the advances in the omics field improve these pathways especially due to the discovery of biomarkers associated with the tumor’s stages.^177^ Carcinoembryonic antigen (CEA), cytokeratin fragment 21–1 (CYFRA21–1), circulating tumor DNA (ctDNA), microRNA (miRNA), DNA methylation, DNA mutations (EGFR, K-RAS, and p53), matrix metallopeptidase 9 (MMP-9), and vascular endothelial growth factor (VEGF) are examples of biomarkers expressed or released from lung cancer cells in trace levels.^178^ Conventional biochemistry or immunology methods apply some of these molecules to detect lung cancer cells, however, these techniques are complex, expensive, and in some cases are not sensitive enough for low-level concentration detections.^179^ Furthermore, methods based on spectroscopy techniques are affected by sample turbidity or interference from absorbing and fluorescing compounds, highlighting the field’s challenges and the need for new detection technologies for these biomarkers.^180^

A cutting-edge method to detect those biomarkers is biosensors, an analytical device that can translate the biological recognition into a physical signal. Some techniques such as electrochemical impedance spectroscopy (EIS), voltammetry, field-effect transistors (FETs), surface enhancement Raman spectroscopy (SERS), surface plasmon resonance (SPR), and fluorescence methods are sensing platforms used to detect lung cancer cells. In the case of electrochemical biosensors, it involves monitoring changes in an electrical signal due to an electrochemical reaction that occurs at an electrode interface, usually because of an imposed potential, current, or signal frequency variation. Some biomarkers are electroactive molecules that can suffer a redox reaction. In other cases, there is no generation of electroactive molecules which makes necessary the indirect monitoring using an electrochemical probe, as in the case of antigen–antibody recognition.^181^ Electrochemical biosensors exhibit several advantages over other detection methods, including increased assay speed, flexibility, simultaneous measurement of various analytes, portability, ease of use, and cost-effectiveness.

Immunosensors to detect the p53 protein, one of the responsible for the regulation of gene expression of cancer cells due to its high concentration in the tumor environment, are of upmost importance. Under cellular stress conditions such as DNA damage, hypoxia, oxidative stress, and mutations, p53 protein is found in higher concentrations and can modulate cellular functions and contribute to genetic maintenance.^182^ In this context, an impedimetric biosensor was developed to detect anti-p53 and anti-p51 antibodies using quantum dots.^183^ EIS measurements showed that the impedimetric biosensor reached a limit of detection (LOD) in the range of 3.3 × 10^–20^ M and a linear range from 10^–10^ to 10^–20^ M, showing high selectivity and sensitivity, similar to other biosensors developed to the same biomarker. Another biosensor based on graphene and functionalized with anti-p53 DNA sequence was developed.^184,185^ The biosensors showed a limit of detection in the order of ppb, like the values obtained by nanosensors to detect miRNA-155 based on Metal–Organic Framework (MOF) material that showed detection limits of 0.038 ppb.

**Figure 7:**
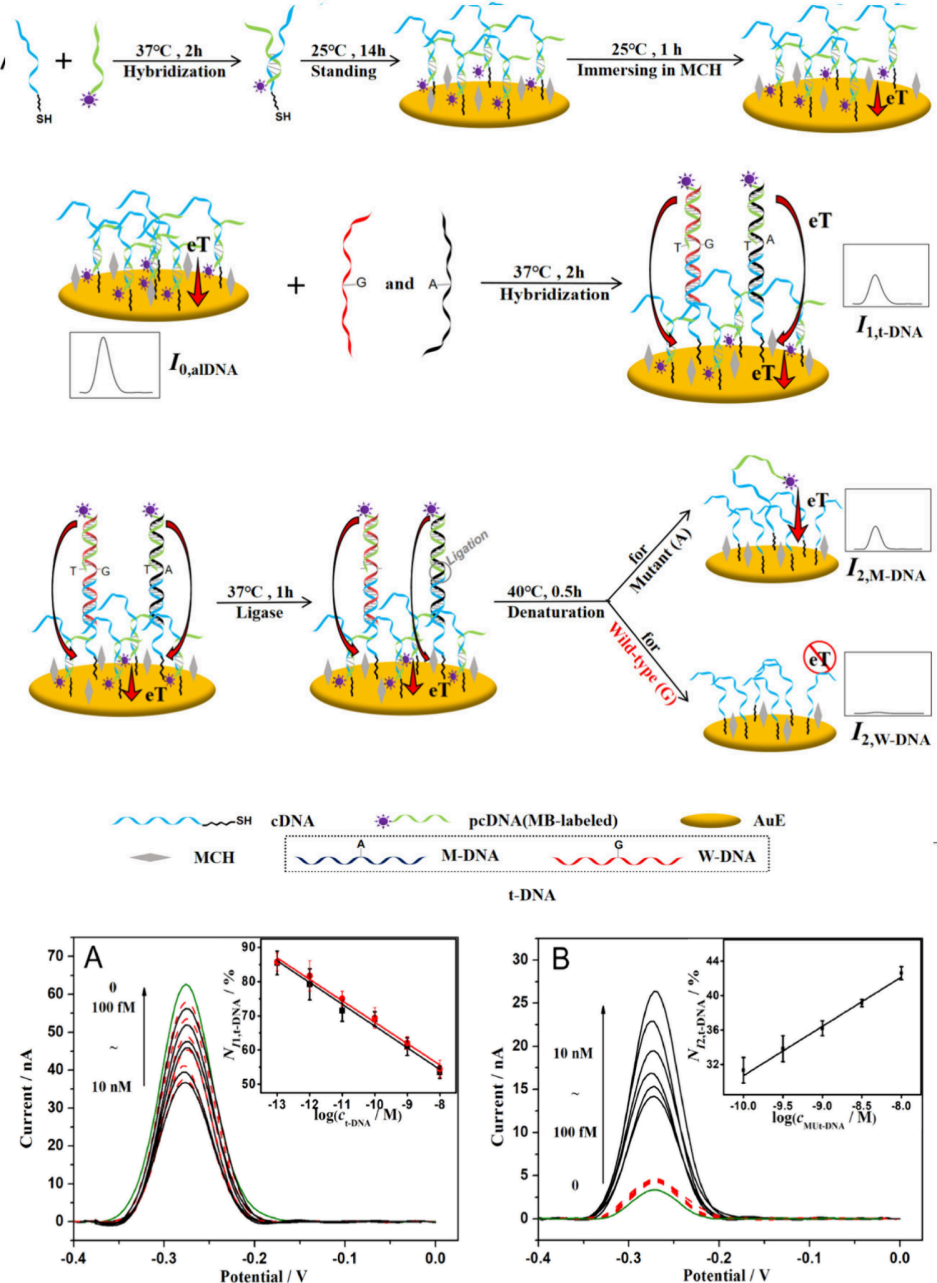
Schematic representation of the formation of the alDNA sensing surface (A) and the detection mechanism of the t-DNA (B) and M-DNA (C) and CV measurements. Reproduced with permission from ref (187). Copyright 2019 Elsevier B.V.

Among the biomarkers with regulatory functions, the KRAS gene is of paramount importance in cellular signal transduction. Activating mutations in the KRAS gene can lead to continuous activation of the cell surface receptor EGFR, resulting in uncontrolled cell growth.^178^ Detection and monitoring of these mutations are of great significance in the field of cancer, as they are associated with poor prognosis and resistance to targeted therapies. For this purpose, electrochemical methods using biosensors have been developed for the specific detection of point mutations in the KRAS gene. Techniques such as cyclic voltammetry, differential pulse voltammetry, and electrochemical impedance spectroscopy have been employed in these studies. Additionally, square wave voltammetry has proven to be an effective technique for the detection of specific point mutations, such as KRAS G12D and G13D (Figure 7).^186,187^

**Figure 8:**
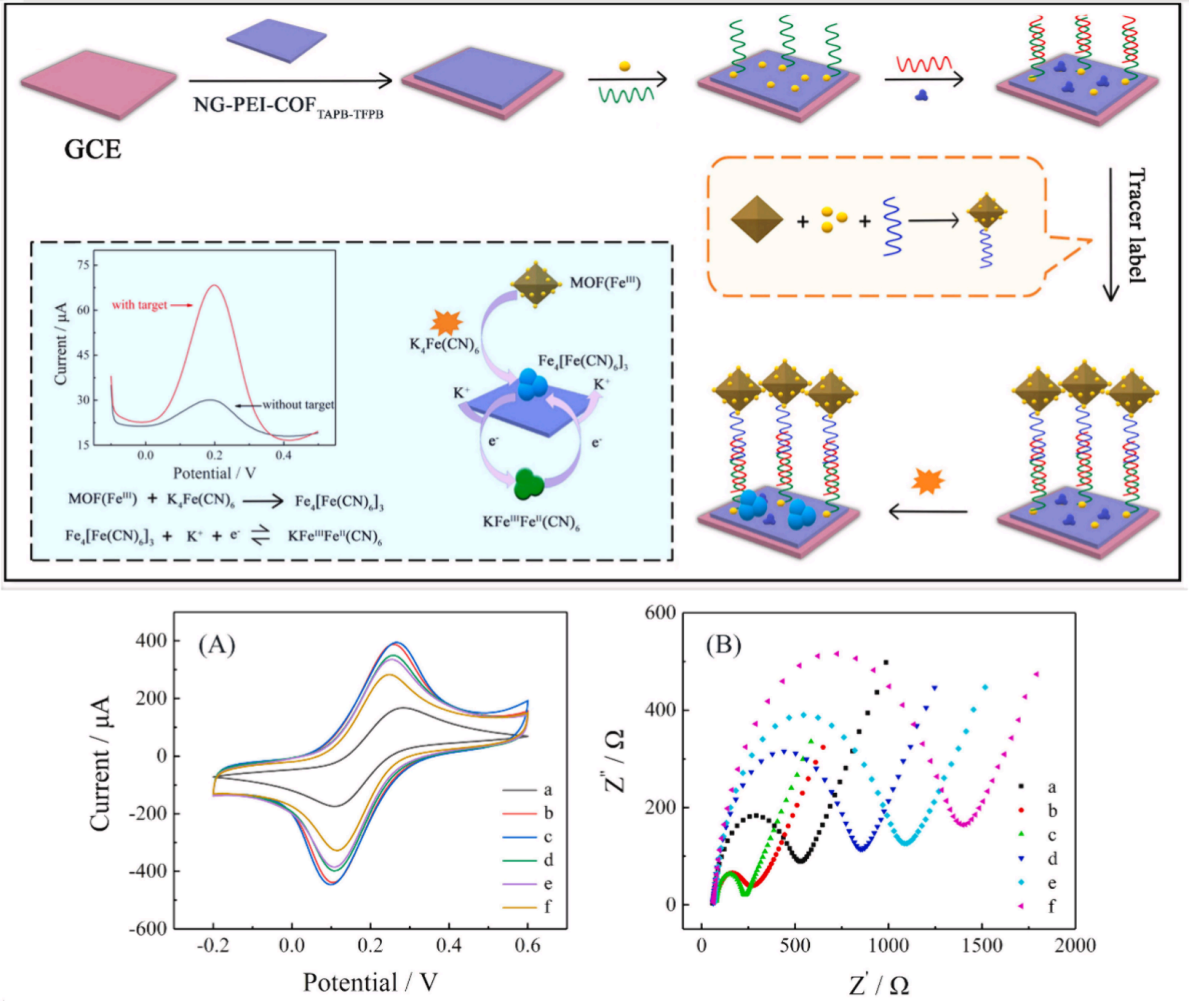
Schematic diagrams of the preparation process of the electrochemical biosensor for ctDNA detection and cyclic voltammetry (CV) responses. Insets (A) and (B) represent the cyclic voltammetry and electrochemistry impedance spectroscopy responses for each modification step of biosensor construction in 0.1 M KCl containing 5 mM K_3_[Fe(CN)_6_]/K_4_[Fe(CN)_6_]. (a) Bare glassy carbon electrode; (b) nitrogen-doped graphene–polyethylenimine–covalente organic framework–glassy carbon electrode; (c) gold nanoparticles/nitrogen-doped graphene–polyethylenimine–covalente organic framework–glassy carbon electrode; (d) capture probe/gold nanoparticles/nitrogen-doped graphene–polyethylenimine–covalente organic framework–glassy carbon electrode; (e) bovine serum albumin/capture probe/gold nanoparticles/nitrogen-doped graphene–polyethylenimine–covalente organic framework–glassy carbon electrode; (f) target probe/bovine serum albumin/capture probe/gold nanoparticles/nitrogen-doped graphene–polyethylenimine–covalente organic framework–glassy carbon electrode. Reproduced with permission from ref (167). Copyright 2019 Elsevier B.V.

Circulating tumor DNA (ctDNA) is a biomarker useful to detect lung cancer, however, there are several difficulties in its analysis resulting from characteristics such as low half-life, low concentrations, and small size of the molecule.^178^ To overcome these challenges CRISPR/Cas12a technology was used to allow their recognition of circulating tumor DNA. Together with MB/Fe3O4@COF/PdAu nanocomposite, the researchers achieved to detection of ctDNA with a low limit of detection of 3.3aM as a result of the signal amplification improvement using the nanomaterial^188^ (Figure 8). Other biomarkers can be applied for the detection of lung tumor cells, such as circulating tumor DNA (ctDNA). An electrochemical biosensor modified with a nickel-catecholate-carbon black metal–organic framework (Ni-CAT-CB) along with gold NPs and a DNA probe showed that as the analyte concentration increased, there was a decrease in current, enabling a LOD of 0.32 fM for ctDNA.^189^

Other biomarkers classified as genetic, or epigenetics are microRNAs (miRNAs). These molecules consist of a small number of nucleic acid pairs and play a significant role in cancer detection due to their high concentration in bodily fluids.^190,191^ Two interesting studies demonstrated the detection of miRNA-21 for lung cancer diagnostics. Both studies employed the electrochemical technique while varying the concentration of the biomolecule on different transducer platforms. One study applied a Zn(TCPP) PET-RAFT-based transducer platform, while the other used a hybrid nanocomposite based on graphene, gold NPs, and conducting polymers. The authors reported LOD values of 4.48 aM and 1.24 fM respectively, indicating high sensitivity.^192,193^

The employment of plasmonic NPs to improve electrochemical response in biosensors has been reported for the sensitive detection of carcinoembryonic antigen (CEA) and epidermal growth factor receptor (EGFR) in protein-based lung cancer analysis.^194^ Multilayered 1D-biosensors were explored to enhance the sensitivity and selectivity of the detection. These plasmonic biosensors offer improvement in the accuracy and efficiency of identification of CEA and EGFR biomarkers, aiding the diagnosis and monitoring of lung cancer.^195^ One of the most cited biomarkers in the literature for lung cancer detection is the cytokeratin 19 fragment 21–1 (CYFRA21–1).^196^ A plasmonic biosensor based on MoS2 modified with carboxyl was used to amplify the signal. The biosensor exhibited detection limits of 0.05 pg/mL, which was lower when compared to the ELISA technique (LOD = 0.60 ng/mL), with a quantitation range of 0.05 pg/mL to 100 ng/mL.^197^

Recently, extracellular vesicles (EVs) have been shown as attractive receptors to detect lung cancer cells due to their association with biomarkers for early diagnosis of lung cancer, and the possibility to improve diagnosis. These EVs play an important role in the physiological and pathological process due to their formation by an endosomal route. Advances for EVs RNAs analysis have been discussed with emphasis on sensors and microfluidics due to their small size from 30 to 150 nm, and the possibility of miniaturization of the diagnosis based on liquid biopsy.^198^ Some studies evidenced the combination of CRISPR and biosensor technologies to improve specificity and sensitivity. It has enabled the detection of the EGFT protein expressed in A549 exosomes in concentrations lower than those detected by the ELISA method, with LOD of 2 × 10–10 exosomes/mL, and managed to differentiate cancer cells of healthy cells.^199^ Fluorescence biosensors are the most used method to improve this type of detection, revealing impressive results such as a sensitivity of 161 fM, and high specificity against mismatched sequences, especially for the determination of exosomes-derived miRNA-21.^200^ Some examples of electrochemical biosensors to detect biomarkers related to cancer cells can be found in Table 3.

**Table 3:** Examples of Electrochemical Biosensor Devices Developed to Early Detect Cancer Cells

sensor	sample	analyte	electrochemical method	limit of detection	linear range	reference
dhDNA (Fc-AP-21/MB-HCP) onto GCE	human plasma	miRNA-141	SWV	0.89 fM	2.0 to 105 fM	(201)
		miRNA-21		1.24 fM		
GO–CS/PVP-AuNUs onto GCE	human plasma	miRNA-141	SWV	0.94 fM	2.0 to 5.0 × 105 fM	(202)
AuNPs/Ni-catecholates/carbon black/polarized pencil graphite electrode (AuNPs/Ni-CAT/CB/PPGE)	serum samples like liquid biopsy	ctDNA from EGFR 19 Dels for NSCLC	DPV	0.32 fM	1.10–15 to 1.10–6 M	(203)
PER-CRISPR/Cas14a	synthetic samples	ctDNA EGFR L858R	DPV	0.34 fM	1 fM to 1 μM	(204)
alDNA sensor	human serum sample	KRAS point mutation level from M-DNA	SWV		100 pM to 10 nM	(187)
g-G-AuNP-dsDNA	synthetic sample	anti-p53 antibody	CV and EIS	0.6 fM	0.1 ng/L to 0.1 μg/L	(185)
SnO2-QD-Au modified with DNA onto AuE	synthetic sample	anti-p53 antibody	EIS	3.2 aM	1.10–6 to 1.10–20 M	(205)

Hairpin-structured DNA (dhDNA); thiolated methylene blue-labeled hairpin capture probe (MB-HCP); ferrocene-modified anti-miRNA-21 DNA probe (Fc-AP-21); square-wave voltammetry (SWV); glassy carbon electrode (GCE); graphene oxide-chitosan@polyvinylpyrrolidone-gold nanourchin (GO–CS/PVP-AuNUs); nonsmall cell lung cancer NSCLC; tricatecholate, 2,3,6,7,10,11-hexahydroxytriphenylene with Ni(II) into metal–organic frameworks is termed Ni-catecholates (Ni-CAT); differential pulse voltammetry (DPV); epidermal growth factor receptor (EGFR) mutation L858R in circulating tumor DNA (ctDNA) (ctDNA EGFR L858R); primer exchange reaction (PER); clustered regularly interspaced short palindromic repeats (CRISPR); associated nucleases (Cas14a); mutante DNA (M-DNA); anchor-like DNA electrochemical sensor (alDNA); graphene-gold NPs composite thin film (g-G-AuNP-dsDNA); cyclic voltammetry (CV); electrochemical impedance spectroscopy (EIS); gold electrode (AuE).

All these diagnosis technologies are possible due to the manipulation of nanomaterials at the molecular level which improves sensibility due to their unique conductive properties. Indeed, the miniaturization and portability of these devices open new possibilities for the development of wearable and implantable biosensors. Unfortunately, some disadvantages like the stability of the biomolecule at the electrode surface and the lifetime of these systems have hampered the commercial application of these devices.

## 7 Clinical Applications of Nanotechnology in Lung Cancer Diagnosis and Treatment

There are currently several nanoformulations available on the market that are clinically used for tumor diagnosis and treatment. These nanoformulations have unique properties that make them ideal for delivering drugs and imaging agents to cancer cells.^206^ They can be designed to accumulate specifically in tumor tissues, thus sparing healthy cells from damage.

Paclitaxel (PTX) is a drug derived from taxanes that acts by interrupting microtubule dynamics, interfering with the G2 mitotic phase, and inhibiting mitosis. PTX is widely used in therapeutic regimens for NSCLC and off-label for SCLC. Due to its lipophilic nature, PTX (Taxol) requires the use of Cremophor-EL (a nonionic surfactant) and ethanol to increase its solubility. However, these components are extremely toxic and can cause hypersensitivity, neutropenia, and neurotoxicity. To overcome these limitations and improve PTX pharmacokinetics, the pharmaceutical industry has launched several surfactant-free nanotechnology-based formulations: Examples are the PTX NPs bound to albumin (nab-paclitaxel or Abraxane), polymeric micelles (Genexol-PM, NK105, Apealea), and liposomes (Lipusu). All these formulations have been clinically approved for the treatment of solid tumors.^173,207^

Nab-PTX allows for the administration of higher doses in shorter periods. It is a NPs that, after intravenous administration, is rapidly broken down into complexes bound to albumin, which mediates PTX transcytosis, internalizing the cell through caveolin-1 protein. Another advantage of nab-PTX, compared to Taxol, is that PTX tissue distribution is faster, and drug clearance is higher.^208^ Another clinically available nanomedicine is Genexol-PM, a formulation composed of micelles based on monomethoxy polyethylene glycol-*block*-poly(D, l-lactide) (mPEG–PDLLA), loaded with PTX. Genexol-PM has the advantage of eliminating the need for an albumin donor, as well as encapsulating PTX within nanostructures, protecting the drug, and further improving its pharmacokinetics compared to nab-PTX, which dissociates rapidly after intravenous administration. Additionally, Genexol allows for higher doses with less hypersensitivity than Taxol.^173^

Lipusu (Sike Pharmaceutical Co. Ltd.) was approved by the State Food and Drug Administration of China. It is the first PTX-loaded liposome injection that entered the clinical market in China in 2006. The advantage of Lipusu is its significant capacity to reduce toxicities.^207^ Yang et al.^209^ studied the cytotoxic effects and antitumor activities of Lipusu and found that it had the same *in vitro* and *in vivo* effect with less toxicity compared to Taxol at the same dosage.

Several other clinical studies based on nanotechnology for the treatment of lung cancer exist, as shown in Table 4. These studies explore the use of various NPs and drug-delivery systems for targeted therapies, including liposomes, polymeric NPs, and nanodiamonds. By using nanotechnology in cancer treatment, drugs can be delivered specifically to cancer cells, reducing the potential for harm to healthy cells and improving the efficacy of treatment. With ongoing research and development in the field of nanotechnology, the potential for improved treatment options and outcomes for patients with lung cancer continues to grow.

**Table 4:** Nanoplatforms Approved and in Clinical Phase for Treatment in Lung Cancer

nanomedicine	nanosystems types	indication	status	reference
ALB-stabilized paclitaxel nanoparticle (ABI-007)	NPs	Stage IV NSCLC	Completed	NCT00077246
Irinotecan liposome injection (ONIVYDE)	Liposome	SCLC	Active, not recruiting	NCT03088813
PTX liposome	Liposome	Advanced NSCLC	Active, not recruiting	NCT02996214
TUSC2-NPs	NPs	Stage IV NSCLC	Active, not recruiting	NCT01455389
PEGylated liposomal doxorubicin and carboplatin	Liposome	NSCLC	Unknown	NCT01051362
Irinotecan hydrochloride liposome injection	Liposome	SCLC	Recruiting	NCT04381910
Encapsulate by nonviral lipid NPs/osimertinib	Lipid NPs	Phase IV NSCLC	Not yet recruiting	NCT04486833
Carboplatin and Paclitaxel ALB-stabilized nanoparticle formulation	NPs	Lung cancer	Completed	NCT00553462

Overall, nanoformulations represent a promising strategy for the diagnosis and treatment of cancer. They have the potential to improve drug efficacy and reduce side effects, and their unique properties make them ideal for targeting specific tissues in the body. As research in this field continues to evolve, more clinically approved nanoformulations will likely become available, providing new hope for cancer patients.

The current state of cancer diagnosis is limited, with only one study in the field of diagnostics being developed using nanotechnology. A device created by the Institute of Bioengineering and Nanotechnology utilizes a microsieve membrane filter to effectively isolate circulating tumor cells from a blood sample, providing a recovery rate of over 85% in just 10 min (NCT04254497). With the increasing incidence of lung cancer, there is a critical need for early detection and diagnosis to improve patient outcomes.

## 8 Recent Progress and State-of-the-Art Nanosystems for Lung Cancer Therapy

In addition to all the advances in the development of nanocarriers for drug delivery discussed until now, nanotechnologies can also be associated with a physical stimulus as the electromagnetic field, to make these systems even more efficient. Some particles, especially metallic ones, such as gold-based NPs, can be stimulated by light inducing localized heat or generating ROS species to kill cancer cells in a specific way. Theranostics that use light energy to provide cancer cell death represent a minimally invasive treatment method that offers efficacy with minimal side effects compared to other conventional cancer therapies.^210−212^ Photoactive agents delivered together with the active drug can participate in the biological pathways to destroy the cancer cells through hyperthermia or photochemical effects, known as photothermal therapy (PTT) and photodynamic therapy (PDT), respectively.^212−214^

To achieve effectiveness in cancer application, studies have shown numerous protocols to associate standards oncology drugs with photoactive agents (such as graphene, carbon dots, plasmonic NPs, transition metal chalcogenides, and oxides) that can improve both PTT and PDT therapies in a synergic way.^214,215^ Moreover, with the aid of a power-adjustable laser irradiation, it is possible to target the tumor, minimizing the potential damage to surrounding healthy tissues.^212,216^ Despite many advances in phototherapy techniques, several challenges persist, which include minimal damage to healthy tissues especially to lung tumors, and the limited depth of penetration of the laser.^217^

The use of plasma membranes derived from tumor cells is an extremely relevant strategy that has been gaining attention.^210,218^ Considered one of the most recent advances in nanobioengineering, the surface modification of NPs using cell membranes has brought a new paradigm for nanomedicine.^219^ A bioinspired and biomimetic strategy can simulate the function and behavior of natural cells. This strategy promotes the accumulation of nanostructures in the tumor microenvironment, in addition to providing a camouflage to evade the immune system. This strategy avoids the need to artificially recreate the cell membrane surface.^220^ Such systems based on cell membranes have diverse therapeutic applications, especially in cancer.

**Figure 9:**
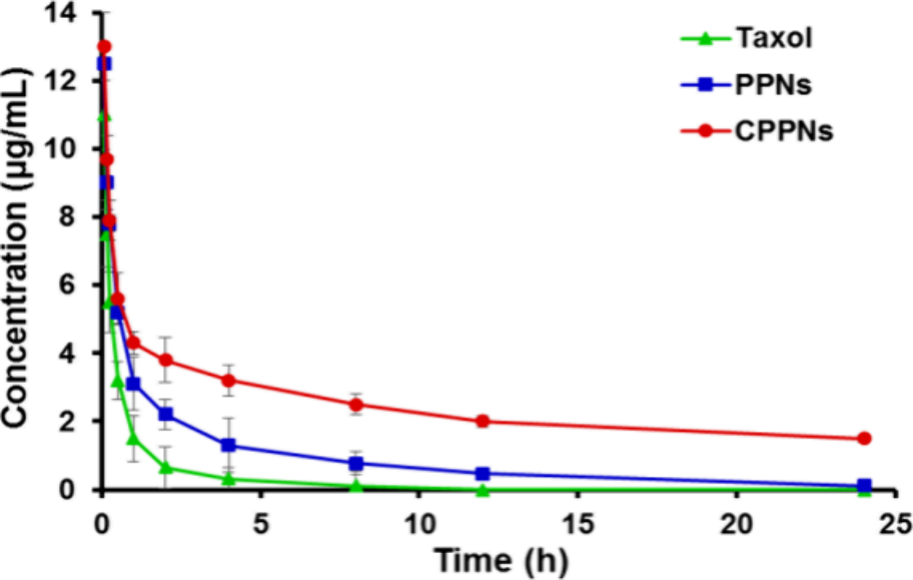
Plasma concentration time profile of Taxol, polymeric NPs, and biomimetic NPs in rats (10 mg/kg). Reproduced with permission from ref (221). Copyright 2016 WILEY-VCH Verlag GmbH & Co. KGaA, Weinheim.

Recently, Sun et al.^221^ reinforced that polymeric NPs coated with breast cancer cell membranes (4T1) (CPPNs) were able to remain longer in the bloodstream and favor specific targeting for tumor cells. The similarity between the surface of the nanocarriers and of the tumor cells, the so-called, homologous adhesion contributed to the systems being recognized and internalized.^221^ The results revealed that CPPNs were able to improve pharmacokinetic properties over previous generations of paclitaxel (PTX)-loaded polymeric NPs (PNPs) and taxol. This improvement was achieved by leveraging natural cell surface features to evade clearance by immune cells, as evidenced by serum drug concentration measurements (Figure 9).

Biomimetic PLGA-based NPs coated with tumor cell membranes and combined with immunological adjuvants have been developed by Kroll et al.^222^ as cancer vaccines. The NPs were presented as antigens, and when combined with immunostimulatory adjuvants (CpG oligodeoxynucleotide 1826), they induced the secretion of pro-inflammatory cytokines by immune cells *in vitro*. The systems not only incorporated a wide range of immune cells but were also able to improve the maturity of dendritic cells and the overall survival of murine models (60%) for five months.^222^ Poly(lactic-*co*-glycolic acid) (PLGA) is a proven safe synthetic polymer, certified by the FDA and the European Medicines Agency (EMA), that has been widely used in clinical practice.^223^ The choice of PLGA as the base for NPs production is due to its recognized biocompatibility, high stability, and nontoxicity. In the physiological environment, PLGA undergoes hydrolysis, breaking down into two monomeric units (lactic acid and glycolic acid) that are easily metabolized by the human body through the Krebs cycle and then easily eliminated as carbon dioxide and water, thus representing no toxicity to patients.^224^

## 9 Nanotoxicology

The same properties that enable NPs to be useful in lung cancer treatment may contribute to their potential toxic effects. The use of exogenous NPs is limited by their potential toxicity, which depends on several factors such as shape, morphology, size, surface charge, biodegradability, biocompatibility, and pharmacokinetics.^225−227^ The use of a safe-by-design (SbD) strategy is essential to improve the efficacy of these nanotechnologies, which means high efficiency with low adverse effects. Identifying and minimizing the risks or even eliminating them during their development is the basis of SbD strategies and it is essential to allow clinical translation.^228,229^

Nanotechnologies applied to lung cancer disease should be evaluated in the same way as new chemical drugs that include pharmacokinetic, pharmacodynamic, toxicology profiles, and efficacy in clinical trials. However, due to the complexity of the physicochemical characteristics of NPs, it has been almost mandatory the modification of standard toxicology tests.^228,230^ Currently, even with the advances in nanotoxicology studies and the regulatory agencies observing these advances, there is a lack of established rules and regulations for the testing of NPs-based cancer therapies which include functional testing and safety evaluation. Aggregation/agglomeration behavior, NPs–NPs interactions, adsorption of proteins, and immunological responses in cells are crucial pieces of information to achieve nanotechnology efficiency for lung cancer.^231^

To extend the circulation time and avoid opsonization, some studies highlight that NPs must be smaller than 100 nm, but it is not a consensus. NPs bigger than 200 nm can be filtered by the spleen and liver, while 10 nm NPs can rapidly pass through renal filtration.^232^ In terms of surface properties, the most biocompatible materials are polymers, liposomes, and proteins that should decrease any possible toxic effects. However, some studies revealed that even for biocompatible polymers bearing a positive charge, the NPs may interact with pulmonary cells differently, due to the electrostatic interactions occurring with pulmonary surfactants, revealing the need to better understand how these surfactants affect the uptake pathways of NPs into cells which may help in reducing the side effects.^233,234^ On the other hand, it has been established that NPs with neutral surface charge (especially the PEG-coated ones) exhibit longer circulation time and less uptake by the mononuclear phagocyte system, due to the decreased opsonization.^235,236^ In the absence of this biocompatibility, NPs may disrupt cell metabolism such as oxidative stress and ROS generation, which cause toxic effects, especially the heavy metals-containing NPs. For example, grade III–IV toxicities were observed in patients with advanced NSCLC and demonstrated favorable antineoplastic results with Genexol-PM in combination with gemcitabine in a Phase II trial.^237^

Polymeric NPs have been extensively used in drug delivery, as discussed before, due to the possibility of encapsulating high concentrations of hydrophobic actives, prolonging the circulation time, and delivery at the target site, reducing treatment side effects.^238−240^ Despite the latter advantages, it is important to decrease the NPs doses in short intervals of time to not affect the cellular functionalities, cell cycle, disruption of mitochondrial membrane potential, and cell viability of organs such as liver or pancreatic beta cells. It is essential to investigate the *in vivo* toxicity and biodistribution of NPs to observe high doses in the tumor microenvironment, and systemic distribution. Some studies revealed that polymeric NPs administered as aerosol may have the potential to reduce systemic toxicity.^239^

*In vivo* studies are important to elucidate the biodistribution, effectiveness, and safety, besides the pharmacokinetics. However, disparities between the efficacy results from preclinical models and clinical trials represent a concern, even with the use of genetically modified mice.^241,242^ The literature suggests that if an animal model could replicate all heterogeneity and anatomical histology of human cancer malignancies, the EPR and NP permeation in metastatic tumors should be elucidated. The clinical translation of nanotechnologies not only to lung cancer but to other cancer types has been threatened by a lack of comprehension of the complex structure of the biological pathways,^166,243^ so before NPs are approved for such treatments, they should be tested in terms of their safety and efficacy in clinical trials.

## 10 Conclusions and Outlook

Lung cancer remains a challenge within the realm of oncology, characterized by late-stage diagnosis and resistance to conventional treatments. Nanotechnology has ushered in a new era, offering innovative nanomaterials with the potential to precisely target cancer cells while sparing healthy tissues. This review explored the aspects of nanomedicines in lung cancer treatment, capitalizing on their unique physicochemical properties. The assessment of these nanotechnologies follows a rigorous evaluation process like that applied to chemical drugs, which includes considerations of their pharmacokinetics, pharmacodynamics, toxicology, and clinical effectiveness. However, due to the unique characteristics of NPs, standard toxicological testing methods require modifications to accommodate their intricacies.

Despite the extensive research efforts, the full potential of nanomedicine has yet to be realized. Clinically approved therapies remain limited, despite the bustling activity in this field. Clear fluctuations in confidence within this maturing field are evident; however, nanomedicine currently stands at a pivotal juncture as more researchers discard old paradigms in favor of emerging concepts. This paradigm shift aligns with the growing knowledge derived from other fields such as molecular biology and immunology.

Throughout this comprehensive review, we have addressed the multifaceted challenges posed by lung cancer and its complex tumor microenvironment. We have also provided an overview of recent advancements in nanoplatforms designed for early diagnosis and treatment. The development of effective therapeutic strategies demands a profound understanding of the disease, encompassing clinical outcomes, the physicochemical attributes of nanomaterials, nanobio interactions, nanotoxicity, and regulatory compliance to ensure patient safety. Our exploration extends beyond the realm of lung cancer, offering insights applicable to a wide spectrum of cancer types and oncological contexts. We advocate for innovative approaches that hold the potential to significantly enhance patient outcomes and overall quality of life. Nanotechnology stands poised to reshape the landscape of cancer management, instilling newfound hope for both patients and healthcare practitioners alike.
